# Subjective versus objective sleep in men with Klinefelter syndrome

**DOI:** 10.1186/s13023-023-02822-2

**Published:** 2023-09-01

**Authors:** K. W. Fjermestad, R. R. Finnbakk, A.-K. Solbakk, C. H. Gravholt, R. J. Huster

**Affiliations:** 1 Department of Psychology, University of Oslo, Forskningsveien 3a, 0373 Oslo, Norway; 2 Frambu Resource Centre for Rare Disorders, Siggerud, Norway; 3 Department of Molecular Medicine, Aarhus University Hospital, Aarhus, Denmark; 4 Department of Endocrinology and Internal Medicine, Aarhus University Hospital, Aarhus, Denmark; 5 Department of Clinical Medicine, Aarhus University, Aarhus, Denmark; 6 RITMO Centre for Interdisciplinary Studies in Rhythm, Time and Motion, University of Oslo, Oslo, Norway; 7 Department of Neurosurgery, Oslo University Hospital, Oslo, Norway; 8 Department of Neuropsychology, Helgeland Hospital, Mosjøen, Norway; 9 Sleep Unit, Department of Otorhinolaryngology/Head and Neck Surgery, Lovisenberg Diaconal Hospital, Oslo, Norway

**Keywords:** Klinefelter syndrome, 47XXY, XXY, Sleep, Mental health, Lived experience

## Abstract

**Objectives:**

To investigate sleep among men with Klinefelter syndrome (KS).

**Method:**

We compared the sleep domains latency, disturbance, and efficiency in 30 men with KS (*M* age = 36.7 years, *SD* = 10.6) to 21 age-matched non-KS controls (*M* age = 36.8 years, *SD* = 14.4). Actigraphs were used to objectively measure sleep across 7 days and nights. Participants also completed a sleep diary over the same period, and the Pittsburgh Sleep Quality Index (PSQI).

**Results:**

The mean correlation between the objective and subjective sleep measures was lower for the KS sample (*M r* = .15) than for controls (*M r* = .34). Sleep disturbance was significantly larger in the KS sample, as measured by actigraphy (*p* = .022, *d* = 0.71) and the PSQI (*p* = .037, *d* = 0.61). In regression models predicting sleep domains from KS status, age, educational level, vocational status, IQ, and mental health, KS status was not a significant predictor. Higher age was associated with more actigraphy-measured sleep disturbance. Higher educational level and being employed were associated with better sleep efficiency.

**Conclusions:**

Sleep disturbance may be a particular problem for men with KS and should be measured with complimentary methods.

## Introduction

Klinefelter syndrome (KS) is a sex chromosome disorder affecting 1:660 males [29]. Nevertheless, KS is considered a rare disorder, as only an estimated 25% of cases are diagnosed [5]. KS affects physical, cognitive, and psychological functioning. Physically, the main concerns are related to hypogonadism, reduced testosterone levels, and accompanying infertility. Cognitively, the average intellectual level of men with KS is normal, albeit lower than among non-KS controls, and with higher performance IQ relative to verbal IQ [28]. Impaired executive functioning, as well as poor expressive and impressive language skills have been documented [9, 14, 18]. Psychologically, men with KS report more psychological distress, as well as lower wellbeing and life satisfaction relative to controls [8, 15, 26]. There is a higher risk for poor emotion regulation skills, as well as depression, anxiety, attention deficit hyperactivity disorder, and schizophrenia [28, 30].

Despite the documentation of several risk factors, there are still knowledge gaps regarding KS. Sleep is an important domain with emerging, yet limited, evidence of problems for men with KS. Sleep disturbance is associated with several negative factors such as poor socio-economic status, mental health problems, and neuropsychological difficulties, all of which are prevalent in men with KS [2]. Given the vast amount of documentation of a reciprocal relationship between sleep and various health domains (e.g., [13, 16], it seems evident that there may be particular issues with sleep also for men with KS, due to their many health challenges. However, surprisingly little research has addressed sleep for this group. We know of two studies that have examined sleep in men with KS. The first was a self-report survey with 53 adults which found that men with KS reported poorer subjective sleep quality, more sleep disturbances, more tiredness affecting daytime functioning, and more use of sleep medication compared to Norwegian normative data [10]. These data were collected using the self-reported Pittsburgh Sleep Quality Index (PSQI; [6]. A later study used an objective sleep measure, i.e., actigraphy watches, to measure sleep over seven consecutive nights in 30 men with KS and age-matched controls [9]. Eight sleep domains were measured. The only domain that significantly differed between the groups was duration of night wakes, measured as the combined duration of the wakes after sleep onset. There were no differences between men with KS and controls in the other domains, which were time going to bed and getting out of bed, number of hours spent in bed, number of minutes awake in bed before falling asleep, sleep duration, number of times waking up during the night, or sleep efficiency, i.e., the percentage of time spent sleeping in bed.

Considered together, these studies suggest that there may be discrepancies between subjective and objective sleep measures for men with KS. This is in line with studies from the general population, in which there is a tendency towards little agreement between objective and subjective measured sleep [12, 21]. Nevertheless, there are reasons why further examination of the potential objective-subjective sleep inconsistency for men with KS is warranted. Subjective reports may be particularly difficult for men with KS due to their language problems. Furthermore, because many health issues in KS may be further impacted by disturbed sleep, sleep may be of key importance for clinicans and patients. Disentangling the role of sleep relative to other problem areas for men with KS may represent a clinical challenge, and it is therefore important to identify the most efficient ways of measuring sleep for this group.

In the current study, we build on the actigraphy data collected from men with KS in a previous study [9] and examine these in relation to subjective sleep measures. We proposed three research questions. First, are there significant differences in objective (actigraphy) and subjective (questionnaire, sleep diary) measures of sleep within the KS sample? Since such discrepancies are common in the general population (e.g., [21], we also expected significant differences in the KS sample. Second, are there differences in objective (actigraphy) and subjective (questionnaire, sleep diary) measures of sleep for men with KS compared to non-KS controls? Due to the many psychosocial health difficulties reported in KS, we expected more sleep problems for men with KS than for controls. Third, are the socio-demographic variables age, educational level, vocational status, IQ, and mental health associated with sleep variables, above and beyond KS status? We examined this because the physical, social, cognitive, and psychological difficulties typically affecting men with KS may be associated with both objectively and subjectively measured sleep. Due to the paucity of research on sleep in KS, we explored this research question without a priori hypotheses.

## Methods

### Sample and recruitment

The KS sample comprised 30 men with KS (*M* age = 36.7 years, *SD* = 10.6, range 18 to 60) who were recruited from various non-clinical settings. Most were recruited through the user registry of a national (non-clinical) advisory center for rare disorders. Other participants were recruited at a Klinefelter syndrome user association meeting or got in touch after watching an online video advertisement posted on various websites, including the Klinefelter syndrome user association website and different rare disorders-oriented online forums.

The control sample comprised 21 men without KS (*M* (mean) age = 36.8 years, *SD* = 14.4, range 18–65). Controls were recruited from various settings, i.e., advertisements in local newspapers; an online video advertisement posted on different websites, including the KS user association website and various rare disorders-oriented online forums; and the social network of male KS participants (note that there was only one family relation, i.e., a cousin). Due to the open nature of recruitment (e.g., advertisements), it was not possible to estimate a response rate. See Table 1 for background information regarding the study samples.

**Table 1:** Participant characteristics

	KS (n = 30)	Controls (n = 21)
Highest education (χ^2^ = 8.547, *p* = .014)	*n (%)*	*n (%)*
Secondary school	4 (13.3)	2 (10.0)
High school	20 (66.7)	6 (30.0)
College/University > 2 years)	6 (20.0)	12 (60.0)
Vocational status (χ^2^ = 11.167, *p* < .001)	*n (%)*	*n (%)*
Student	4 (13.3)	1 (5.0)
Currently working	15 (50.0)	19 (95.0)
On welfare benefits	8 (26.7)	0 (0.0)
Other (retired, unemployed)	3 (10.0)	0 (0.0)
Marital status (χ^2^ = 6.111, *p* = .047)	*n* (%)	*n (%)*
Single	16 (53.3)	4 (20.0)
Married/co-habiting	12 (40.0)	15 (75.0)
Divorced/separated	2 (6.7)	1 (5.0)
IQ scores	*M (SD)*	*M (SD)*
Total IQ (*p* < .001, *d* = − 1.31)	98.0	115.8
Verbal IQ (*p* < .001, *d* = − 1.54)	92.2	114.0
Performance IQ (*p* < .05, *d* = − 0.77)	103.0	114.3

*KS* Klinefelter syndrome; *IQ* Intelligence Quotient

### Procedures

The current study is part of a larger trial in which participants went through resting state structural magnetic resonance imaging, electroencephalogram recordings, and neuropsychological testing (data not presented here). On the day of testing, participants completed a sleep questionnaire on site. At the end of the testing day, they were given a Phillips Actiwatch Spectrum Plus watch, with instructions to wear it constantly for the next consecutive seven days. They were also given a sleep diary to complete daily. The Actiwatch and the sleep diary were to be returned in an envelope with prepaid postage after the 7 days. All participants completed the assessment and returned the Actiwatch and the sleep diary.

The study was approved by the Regional committee for medical and health research ethics, and all participants provided written informed consent prior to study participation. Participants took part in a draw for a universal gift certificate (≈ 100 USD; one per sample). They were not compensated in other ways, but travel and accommodation costs were covered, and beverages and food were served during test days. All participants received a written report summarizing their sleep profile, as well as the other domains covered in the survey (i.e., neuropsychological profile). Referrals were made for additional clinical services when indicated. Two such referrals were made, which concerned mental health and neuropsychological issues unrelated to sleep.

## Measures

### Objectively measured sleep

*Actigraphy* Sleep and circadian rhythm were measured with actigraphy using the Actiwatch Spectrum plus (Phillips). The Actiwatch is an actigraph integrated in a small wristwatch casing. Predefined algorithms are used to analyze the recorded raw data from the watch based on measures of motion and ambient light. Actigraphy is commonly used to assess sleep/wake patterns based on periods of activity versus inactivity and light measurements. Participants wore the Actiwatch watch for 7 consecutive days. The 7-day sleep registration was either done Sunday-Saturday or Monday-Sunday.

In the current study, we used actigraphy-generated data for the mean over those 7 days and nights on three sleep domains: (a) sleep latency, measured as the number of minutes the person lay in bed awake between going to bed and falling asleep; (b) sleep disturbance, measured as the combination of number and duration (mins) of night wakes, and (c) sleep efficiency, measured as the percentage of time spent in bed used for sleep (sleep duration/time in bed).

## Self-reported sleep

*The Pittsburgh Sleep Quality Index*** (**PSQI; [6, 24] was used as a measure of subjective sleep. The PSQI comprises 19 items covering seven sleep domains. In the current study, we included the domains that could be compared to actigraphy and sleep diary data. i.e., sleep latency, sleep disturbance, and sleep efficiency. Every domain is rated from 0 (no problems) to 3 (severe problems), with a possible total score range from 0 to 15. Participants rate their sleep based on the last month. In the current study, internal consistency (Cronbach’s α) for the PSQI was α = 0.77 for men with KS, and α = 0.82 for controls.

*The Sleep Diary* [4] was used as an additional measure of subjective sleep. Participants completed the sleep diary, which comprised 10 items, each day for the seven days they wore the Actiwatch. In the current study, we included sleep latency and sleep disturbance. Sleep efficiency cannot be rated based on the sleep diary.

## Socio-demographic variables

Educational level (highest school level) and vocational status (employed or not) were measured with a self-reported background questionnaire developed for this study.

*The Wechsler Abbreviated Scale of Intelligence* (WASI; [31] was used to estimate general intellectual function (i.e., IQ). The WASI comprises four tests. The Vocabulary and Similarities tests provide indices of word knowledge, verbal reasoning, and concept formation (i.e., verbal IQ). Block design and Matrix Reasoning provide indices of the ability to analyze and organize abstract visual stimuli, nonverbal concept formation, perceptual analysis/organization, and abstract reasoning skills for visual stimuli (i.e., performance IQ). The test administrators were a team of advanced psychology students trained by an experienced clinical neuropsychologist.

*The Hopkins Symptom Checklist-90-revised* (SCL-90-R; [7] was used to measure mental health. The SCL-90-R is a self-report questionnaire where 90 mental health symptom items are rated 0 (not at all) to 4 (very much) for the last 7 days. We used the SCL-90-R Global Severity Index as a measure of mental health in the current study. The Norwegian version of the SCL-90-R has documented psychometric properties [25]. In the current sample, internal consistency (Cronbach's α) was excellent for men with KS (α = 0.97) and controls (α = 0.92).

### Data analytic plan

For the within-KS sample comparisons between objective and subjective sleep measures, we used paired sample t-tests. We did not include the sleep disturbance score from the PSQI, as this is measured on a different parameter than the actigraph and the sleep diary, which both measured sleep disturbance as number*duration of night wakes. For the between-KS and control sample comparisons, we used ANOVAs with KS versus controls as the grouping variable. To examine the role of socio-demographic variables for sleep, we ran a series of eight linear regression models, predicting the three sleep domains (latency, disturbance, efficiency) for the three measures (actigraphy, sleep diary, questionnaire) from the following background variables: age, educational level, vocational status, IQ (total, verbal performance), and mental health. The sleep measures were labelled latency/disturbance/ efficiency_Act/Diary/PSQI_, respectively. Group (KS versus controls) was included as a predictor in each of the models. Across variables, the average amount of missing data was 6.4%. Little’s Missing Completely at Random (MCAR) test showed that data were missing completely at random. Missing data were deleted listwise. We conducted all analyses with IBM Statistics SPSS 27.0.

## Results

There were significant differences between the samples in terms of background variables. Compared to controls, the men with KS had lower educational level and IQ (total, verbal, performance), and fewer were working or married/co-habiting (Table 1).

### Differences in subjective and objective sleep measures in KS

The KS sample showed a significant difference between disturbance_Act_ and disturbance_Diary_ (*p* = 0.005, *d* = 0.71), with the actigraphy showing more sleep disturbance (i.e., more frequent and longer-lasting night wakes). There were no within-sample differences between the measures regarding sleep latency or efficiency.

### Correlation analyses

In the KS sample, there was one significant correlation between an objective and a subjective sleep measure, i.e., a medium correlation between efficiency_Act_ and efficiency_Diary_ (Table 2). In the control sample, there were two significant correlations between objective and subjective sleep measures, i.e., a strong correlation between efficiency_Act_ and efficiency_Diary_ and a strong correlation between disturbance_Act_ and efficiency_PSQI_. The mean correlation between the objective and subjective sleep measures was lower in the KS sample (*M r* = 0.15) than for controls (*M r* = 0.34). These findings indicate that there may be less objective-subjective overlap in the KS sample.

**Table 2:** Sleep in men with Klinefelter syndrome and controls

Sleep variable	Actigraphy		Sleep diary		PSQI	
	KS	Controls	KS	Controls	KS	Controls
*M (SD)*						
Sleep latency^a^	34.5 (30.6)	31.7 (20.4)	21.8 (12.3)	17.6 (11.9)	38.4 (45.8)	25.1 (22.8)
Sleep disturbance	91.0 (27.7)	72.7 (22.6)	31.3 (72.5)	18.4 (28.7)	1.5 (0.7)*	1.1 (0.5)
Sleep efficiency^b^	79.3 (8.3)	81.8 (6.3)	–	–	79.2 (17.3)	84.4 (11.3)

*KS* Klinefelter syndrome

^a^Sleep latency is time (in minutes) from getting to bed until falling asleep

^b^percentage of time in bed spent sleeping (not measured with sleep diary)

*Group difference is significant at the p < .05-level

Across the subjective sleep measures, there were three significant correlations in the KS sample and four significant correlations in the control sample. The mean correlation between the subjective sleep measures was *r* = 0.24 in the KS sample and *r* = 0.36 in the control sample. These findings indicate somewhat lower subjective consistency in the KS sample. See Table 3.

**Table 3:** Correlations between subjective and objective sleep measures in Klinefelter syndrome (above diagonal) and controls (below diagonal)

	PSQI			Sleep diary			Actigraphy		
	Late.	Dis.	Effic.	Late.	Dis.	Effic.	Late.	Dis.	Effic.
*PSQI*									
Late.	1	.18	− .55**	.29	.24	− .03	− .08	− .24	− .10
Dis.	.43	1	− .59**	.30	.07	− .05	− .09	.19	− .07
Effic.	− .57**	− .42	1	− .36	− .13	.18	− .06	− .20	.29
*Sleep diary*									
Late.	.85**	.51*	− .55	1	− .12	− .10	− .04	− .15	.00
Dis.	.12	− .06	− .39	.05	1	− .45*	− .23	.08	− .05
Effic.	− .40	− .32	.45	− .23	− .52*	1	− .09	− .32	.38*
*Actigraphy*									
Late.	.41	.39	− .46	.32	− .22	− .28	1	.14	− .76**
Dis.	.17	.27	− .61*	.31	.02	− .45	.37	1	− .36
Effic.	− .46	− .07	.52	− .44	− .10	.55*	− .64*	− .60**	1

Late. = Sleep latency, i.e., time from bedtime to falling asleep (mins). Dis. = Sleep disturbance (i.e., number and duration). Effic. = sleep efficiency

*Correlation was significant at the *p* < 0.05 level

**Correlation was significant at the *p* < 0.01 level

### Between-group sleep differences

We compared sleep latency, sleep disturbance, and sleep efficiency measured by actigraphy, sleep diary, and questionnaire measures in a series of ANOVAs using KS versus controls as the grouping variable. The analyses showed two significant group differences. There was larger disturbance_Act_ in the KS sample than among controls (*F* = 5.59, *p* = 0.022, *d* = 0.71). There was also larger disturbance_PSQI_ in the KS sample relative to the controls (*F* = 4.61, *p* = 0.037, *d* = 0.61). No other between-group differences appeared (all *p* > 0.227).

### Associations between sleep and socio-demographic variables

Two of the eight models predicting sleep domains from participant background variables were significant (Table 4). Higher age was associated with more sleep disturbance_Act_ and explained 25.4% of the variance (*adj. R*^*2*^; p = 0.008). In the model for efficiency_PSQI_, higher educational level and being employed were associated with better sleep efficiency_PSQI_, explaining 19.4% of the variance (*adj. R*^*2*^; p = 0.038). None of the other regression models were significant (all *p* > 0.144, data not shown).

**Table 4:** Sleep domains predicted by socio-demographic variables; significant models

Sleep domain and predictors	*β*	*SE*	*t*	*p*	95% CI for *β*
*Actigraphy-measured sleep disturbance*					
Group	− 8.076	10.649	− 0.758	.453	− 29.654 to 13.502
Age	1.112	0.320	3.474	.001	0.463–1.760
Educational level	− 8.973	4.762	− 1.884	.067	− 18.621 to 0.675
Vocational status	− 8.584	8.830	− 0.972	.337	− 26.476 to 9.308
IQ	0.066	0.308	0.216	.830	− 0.557 to 0.690
Mental health	− 0.061	0.096	− 0.636	.529	− 0.255 to 0.133
*Questionnaire-measured sleep efficiency*					
Group	− 8.678	6.205	− 1.399	.171	− 21.301 to 3.946
Age	− 0.265	0.185	− 1.432	.162	− 0.624 to 0.112
Educational level	5.831	2.804	2.079	.045	0.125–11.536
Vocational status	12.312	5.204	2.366	.024	1.724–22.900
IQ	− 0.138	0.191	− 0.724	.474	− 0.526 to 0.250
Mental health	− 0.070	0.056	− 1.239	.224	− 0.185 to 0.045

Group = Klinefelter syndrome versus controls. *CI* confidence interval; *IQ* intelligence quotient

In summary, most sleep domains were not predicted by the demographic and socio-demographic variables included in the current study. Age, educational level, and vocational status were the only significant predictors.

## Discussion

We aimed to enhance current knowledge about sleep for men with KS. Within the KS sample, there was a significant difference in how much sleep disturbance the participants reported in their subjective sleep diary and how much sleep disturbance the objective actigraphy measure showed. The objective measure showed significantly more sleep disturbance than the sleep diary. We believe the main explanation for this difference is that the actigraph measures micro-wakes and night wakes of very short duration, which participants do not subjectively perceive. There was no difference between the objective and the subjective sleep latency or efficiency measures. We therefore conclude that the correspondence between subjective and objective sleep for men with KS is similar to controls, but that there may be some more sleep disturbance that men with KS do not perceive.

We also examined differences between the KS sample and controls on the sleep domains. We found significant differences on the sleep disturbance domain, both based on the objective measure (actigraph) and the questionnaire measure (PSQI). The differences were of medium size, with more sleep disturbance for the KS sample. This is in line with a previous study of another sample of men with KS which showed that they subjectively reported poorer sleep, both in terms of quality and quantity, relative to normative data [10].

Contrary to our expectation, there were no significant differences between the samples regarding sleep latency or efficiency. Furthermore, KS status did not predict any of the sleep domains. We were surprised by the low number of differences between the KS sample and controls, as we expected that the many health challenges documented for men with KS would include sleep problems. Poorer perceived health is also associated with poorer sleep quality [17]. Men with KS typically report worse health than controls [15]. They also tend to describe inferior physical, emotional, and social functioning [8]. Worries and concerns are usually associated with sleep problems [3]. The small sample size and the fact that our participants were recruited from non-clinical settings may have prevented us from uncovering such associations.

Age, vocational status, and educational level explained variance in a few sleep domains. Several studies have shown increasing sleep problems with increasing age (e.g., [19, 23]. The most likely explanation for why age only predicted one sleep domain in the current study is the small sample size and the fact that none of our participants with KS were above 60 years of age. Positive associations between work status and educational level and sleep are in line with previous research with both clinical and non-clinical populations [3, 20, 22, 23]. These findings are hardly surprising, as most employers expect workers to keep steady schedules, getting to work on time, and leave people more tired and ready for sleep in the evenings. Thus, sleep problems for men with KS clearly need to be assessed and considered in light of their daily routines and employment status. Only half of the KS sample reported to be currently working. At the group level, men with KS have below average socio-economic status compared to non-KS reference groups on several domains, including vocational status [5, 11]. Such patterns need to be kept in mind when evaluating sleep for men with KS.

### Strengths and limitations

A strength of the current study is the use of multiple sleep measures, including an objective actigraph measure over time. Another advantage is the use of a control sample. However, some limitations should be noted. The sample size was small and probably left us underpowered to detect other significant associations. KS being a rare disorder, however, the size of our sample was comparable to the median number of participants of 27 individuals found across 19 reviewed KS studies [28]. Sleep was only registered over seven days. Although this is considered a sufficient time period for actigraphy measures [1], the participants may have been particularly aware of their sleep patterns and tried to adhere to recommendations during that week. A longer assessment period may have prevented or revealed such adherence effects.

The three sleep measures used in the current study conceptualize sleep domains slightly differently, and the two self-report measures are based on different time periods (one week for the sleep diary and one month for the PSQI). This complicates comparisons between the measures. Another limitation concerns the expectation of linearity, which is the basis of the regression models we used. Future studies with larger samples should explore potential non-linear associations between socio-demographic variables and sleep. For example, different predictors may explain who under-reports and who over-reports subjective sleep. We included a large number of predictors for our sample size, and did not correct for multiple comparisons. We did this as a first exploratory study of sleep domains, but caution is warranted regarding the predictor findings, which need to be re-examined in larger samples before firm conclusions can be drawn.

We recruited participants through multiple non-clinical settings, including advertisements. Thus, we could not calculate a response rate and determine how representative the KS sample is. However, the choice of recruitment method also allowed for more diversity among the KS sample than for example clinic only-recruitment would have given.

### Implications and conclusion

The main implication of the current study is that for sleep latency and efficiency, the subjective perception of sleep in men with KS seems to overlap sufficiently with objective sleep measures. These domains also appear similar to controls. For sleep disturbance, there is less overlap and also a significant difference from controls. In contexts where objective measures are not possible, asking patients to complete a sleep diary over at least seven consecutive days gives an indication of sleep that is closer to objectively measured sleep than a retrospective one-time questionnaire. However, although actigraphy is not realistically applicable in many clinical settings due to the associated costs, there are several built-in sleep and activity registrations on smart phones which are commonly used by patients. These could be applied in clinical practice. Sleep should also be considered in light of age, educational level, and vocational status, which all predicted sleep domains.

Sleep research in men with KS is still a relatively uncharted territory. Future research should examine the links between sleep and other variables, like physical complaints and mental health, with larger samples recruited across clinical and non-clinical settings.

## Data Availability

Data are available upon request from the first author. They are not available online in line with the data protection agency that approved the study.
